# Soil invertebrates occurrences in European North-East of Russia

**DOI:** 10.3897/BDJ.8.e58836

**Published:** 2020-11-30

**Authors:** Tatyana Konakova, Alla Kolesnikova, Anastasia Anatolevna Taskaeva

**Affiliations:** 1 Institute of Biology of Komi Scientific Centre of theInstitute of Biology of Komi Scientific Centre of the Ural Branch of the Russian Academy of Sciences, Syktyvkar, Russia Institute of Biology of Komi Scientific Centre of theInstitute of Biology of Komi Scientific Centre of the Ural Branch of the Russian Academy of Sciences Syktyvkar Russia

**Keywords:** occurrence, Lumbricidae, Chilopoda, Diplopoda, Collembola, Staphylinidae, Elateridae, soil, tundra, taiga, mountains, North-East of Europe

## Abstract

**Background:**

The European North-East of Russia is the territory which includes the Nenets Autonomous District, represented by the East European tundra (from Kanin Peninsula to Vaigach Island), Komi Republic with its taiga ecosystems and the Urals (Northern, SubPolar and Polar). Over 20 years of systematic studies of soil fauna in the studied region has resulted in a huge amount of data being accumulated that can be analysed from different positions. Considering that the representation of Russian soil biota data, especially from European North-East of Russia in the GBIF database is not large, our data are of great interest to the scientific world community. The accumulation of such data will solve questions on national and global scales using large arrays.

This study produced a dataset containing information on occurrences on soil invertebrates (Lumbricidae, Chilopoda, Diplopda, Collembola, Elateridae and Staphylinidae) in the European North-East of Russia. The dataset summarises occurrences noted in natural and disturbed forests, tundra and mountain ecosystems.

**New information:**

Data from 196 geo-referenced localities of European North-East of Russia (tundra, taiga and mountains ecosystems) have been collated. A total of 5412 occurrences are included in the resource. The current project surveys 13 species of earthworms, 20 species of millipedes, 246 species of springtails, 446 species of rove beetles and 60 species of click beetles. The diversity of soil invertebrates in the European North-East of Russia has not been fully explored and further exploration will lead to more taxa.

## Introduction

Soils are increasingly recognised as crucial components of ecosystems and biodiversity (Wardle et al. 2004, Bardgett and Wardle 2010) and they represent unique compartments of terrestrial ecosystems by comprising components of the atmosphere, biosphere, hydrosphere and lithosphere. Soil biodiversity supports many terrestrial ecosystem functions (Wall et al. 2012) and delivers important ecosystem services, such as food and fibre production, carbon sequestration and degradation of pollutants. However, the data and information regarding diversity that lives in soil remain insufficiently catalogued and coordinated and this limits our ability to fully assess the key role which soil biodiversity plays in supporting terrestrial systems and ecosystem services (Ramirez et al. 2015).

Databases of specific soil-animal groups, for example, Chilobase, Collembola.org or Lumbricidae and others, offer complete taxonomical lists of the worldwide known species, partly with bibliographic references to the original descriptions, determination keys etc. Unfortunately, none of these databases collects or offers comprehensive background habitat information and distributional data are often not georeferenced or only available at very large and rough spatial scales. Therefore, they do not provide the data necessary for meta-analyses of detailed species’ spatiotemporal distributions or of species’ ecological niche spaces. However, synthesis of global soil invertebrate data would allow many fundamental questions to be addressed relating to ecology, evolution, ecotoxicology and conservation. For example, basic macroecological patterns, such as effects of elevational and latitudinal gradients on diversity, could be examined with such a dataset. The other special interest is in determining the historical forces driving species biogeography. For many species, the chance of winning the battle against global extinction depends on their ability to both live in a range of environments and the ability to track them. During last 5-10 years, one of the most important questions relating to global distributional patterns of soil animals is related to diversity patterns and the abiotic and biotic factors driving global distributions of native and non-native species. The occurrence dataset in GIBF is the first step to understanding species distribution. These data are able to shed light on the occurrence and distribution pattern of rarely-detected species within a given area of interest. Previously, we have already published data on the occurrence of ground beetles (Konakova and Kolesnikova 2020) and sampling datasets of springtails (Taskaeva 2020a, Taskaeva 2020b, Taskaeva 2020c, Taskaeva and Melekhina 2020).

In this data paper, we describe this dataset and make it freely available online for future use.

## General description

### Purpose

The main goal was to describe a dataset on soil invertebrates (Collembola, Lumbricidae, Myriapoda, Staphylinidae and Elateridae) occurrences in European North-East of Russia. The objectives of this study are (i) to include literature data of observations beginning from 1905 and the last 20 years in the study area, (ii) to provide a basis for analysing spatio-temporal changes in biodiversity and landscape in the study area.

## Project description

### Title

Soil invertebrates occurrences in European North-East of Russia

### Personnel

Tatyana Konakova, Alla Kolesnikova and Anastasia Taskaeva

### Study area description

The European North-East of Russia (ENER) is the territory which includes the Nenets Autonomous District, represented by the East European tundra (from Kanin Peninsula to Vaigach Island), Komi Republic with its taiga ecosystems and the Urals (Northern, Subpolar and Polar). The study area is also composed of a diverse set of land parcels of different historical use in agriculture and local activity. There are some enterprises producing paper, oil, coal etc.

The largest part of European North-East of Russia is covered by forests. Spruce forests, which are a zonal type of vegetation, dominate. Following the spruce, pine forests also exist in the study area with birch trees being slightly less in number. Forests formed by fir, larch, Siberian cedar and aspen cover much smaller areas and not all territories of the region.

In the Far North of the region, there is a tundra zone. The vegetation cover here is represented by various types of tundra combined with swamps. Moss tundra are common on the coast of the Barents Sea. To the south, spotted shrub-moss and shrub tundra prevail. In river valleys, thickets of high willows have developed.

The Ural Ridge is represented by the slopes of the Polar, Subpolar and Northern Urals. In mountain conditions, vertical distribution of vegetation stands out: mountain-forest, podholtz, mountain-tundra and a belt of cold deserts. The National Park “Yugyd-Va” and Pechoro-Ilych Reserve, being the zones of ecological, faunistic and floristic interest (UNESKO) and belonging to the national inventory of natural heritage, are situated at the Urals.

The climate of the region is temperate continental with local specificities due to the topography

and mixed land-cover. Towards to the north, it is replaced by the Arctic. The northern part lies in the permafrost zone. The average July temperature in the north is +15°C, in the southern part +18°C. The duration of the summer period is 50-60 days in the north and 80-100 days in the south. Winter is the longest period of the year and lasts 5-8 months (Kolesnikova et al. 2017).

### Design description

The study of the soil invertebrates diversity of terrestrial ecosystems in the ENER is aimed to implement a species inventory including records from the 20 last years of research. Soil invertebrates sampling took place mainly during the summer period. Records were mostly observations and preserved specimens, because the studied territory places a high priority on biodiversity conservation and protection. The standard methods of soil zoology were used.

The results of this study were documented in the Institute of Biology of Komi Scientific Centre of the Ural Branch of the Russian Academy of Sciences.

### Funding

The Ministry of Education and Science of the Russian Federation. Project No АААА-А17-117112850235-2 "Distribution, systematics and spatial organisation of fauna and animals population in taiga and tundra landscapes and ecosystems at the Northeast European Russia"

## Sampling methods

### Study extent

We built our database by compiling occurrences of soil invertebrates species, based on an exhaustive search in published and unpublished authors' sources, as well as from museums of the Institute of Biology Komi SC UB RAS, Syktyvkar State University and the personal collection of K.F. Sedykh.

### Sampling description

Soil and litter dwelling invertebrates were sampled at 196 points evenly distributed over the study area. Three sampling methods were used:

1. Soil extraction (0-10 cm deep, 5-10 cm diameter for mesofauna and 25×25 cm for macrofauna), followed by the Tullgren funnel method over a 7-day period.
2. Manual soil sampling.
3. Pitfall traps or Barber traps were made of plastic pots (10 cm deep, 12 cm diameter), filled with salt water (30% v/v) and formalin (5% v/v) and buried to the rim into the soil.
4. A count with an entomological net.
5. Exhauster method
6. A visual search

All soil invertebrates were identified to the species level using applicable determination keys (Andersson et al. 2005, Costa et al. 2010, Fjellberg 1998, Fjellberg 2007, Herman 2001, Potapov 2001, Kaprus et al. 2016, Vsevolodova-Perel 1997).

On each slide and/or label, the following fields were filled out: “Collection date”, “Locality” (with geographic coordinates), “Habitat”, “Collector name” and “Determined by” (identification).

### Quality control

The data were collected and identified to species by specialists from the Institute of Biology of Komi Scientific Centre of the Ural Branch of the Russian Academy of Sciences. Some Collembola, Lumbricidae, Myriapoda, Staphylinidae and Elateridae specimens were identified by taxonomic specialists from the Severtsov Institute of Ecology and Evolution of the Russian Academy of Sciences, Moscow State Pedagogical University, Institute for Problems of Ecology and Mineral Wealth Use of Tatarstan Academy of Science, Tembotov Institute of Ecology of Mountain Territories of Russian Academy of Science, Institute of the Industrial Ecology Problems of the North, Zoological Institute of Russian Academy of Sciences and the Zoological Museum of Moscow University.

### Step description

In each biotope, 20-30 soil samples were collected. Barber traps were made of plastic pots (10 cm deep, 12 cm diameter), filled with salt water (30% v/v) and formalin (5% v/v) and buried to the rim into the soil. Individuals, that could not be identified in the field, were stored in 95% ethanol for later identification using binocular loupes. Collembola were then slide-mounted on cavity slides and identified to the species level using applicable determination keys (Fjellberg 1998, Fjellberg 2007, Kaprus et al. 2016, Potapov 2001). On each slide, the following fields were filled out: “Collection date”, “Locality” (with geographic coordinates), “Habitat”, “Collector name” and “Determined by” (identification). Some parts of the springtails were kept in alcohol.

## Geographic coverage

### Description

**Figure 1.**
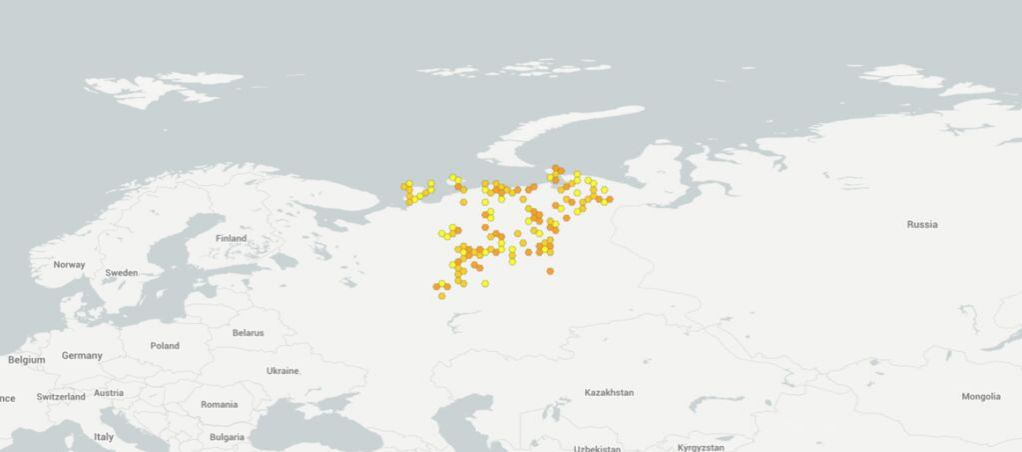
Sampling localities for soil invertebrates in the European North-East of Russia included in the dataset (https://www.gbif.org/dataset/a3f7d0d8-d9e1-42ae-b1f7-d1fc445b931d).

**Figure 2.**
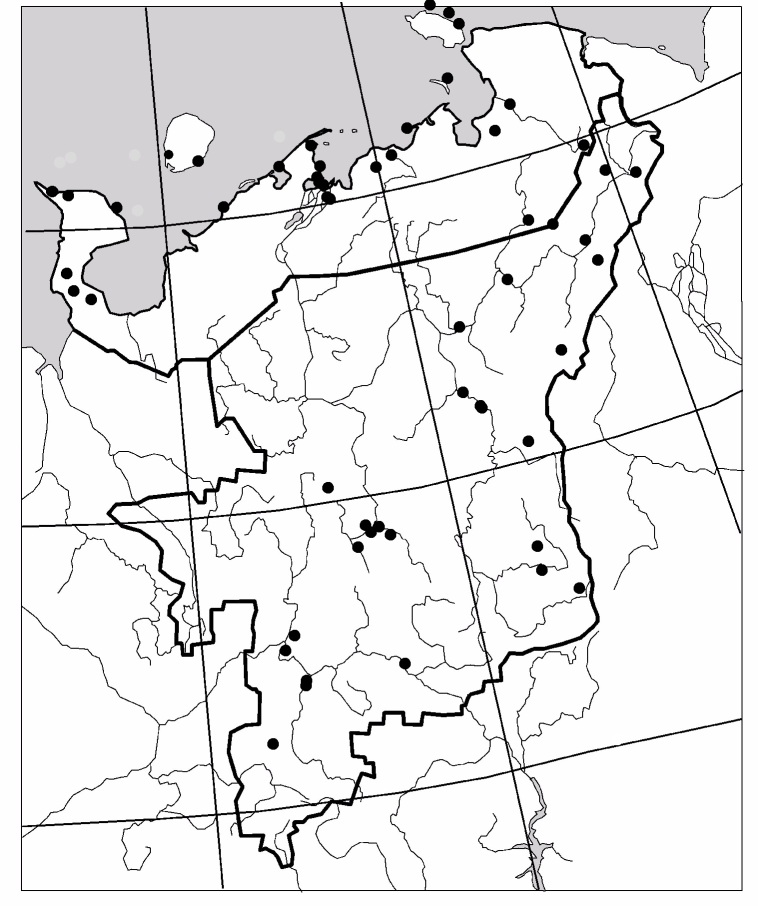
Sampling sites of Collembola in North-East Europe are included in the dataset.

**Figure 3.**
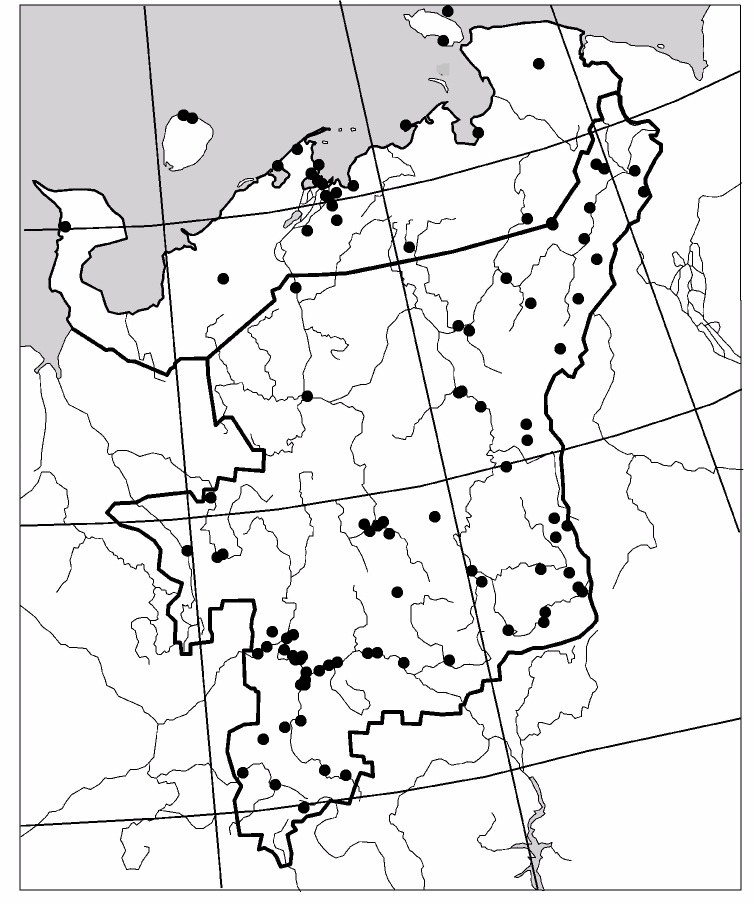
Sampling sites of Lumbricidae in North-East Europe are included in the dataset.

**Figure 4.**
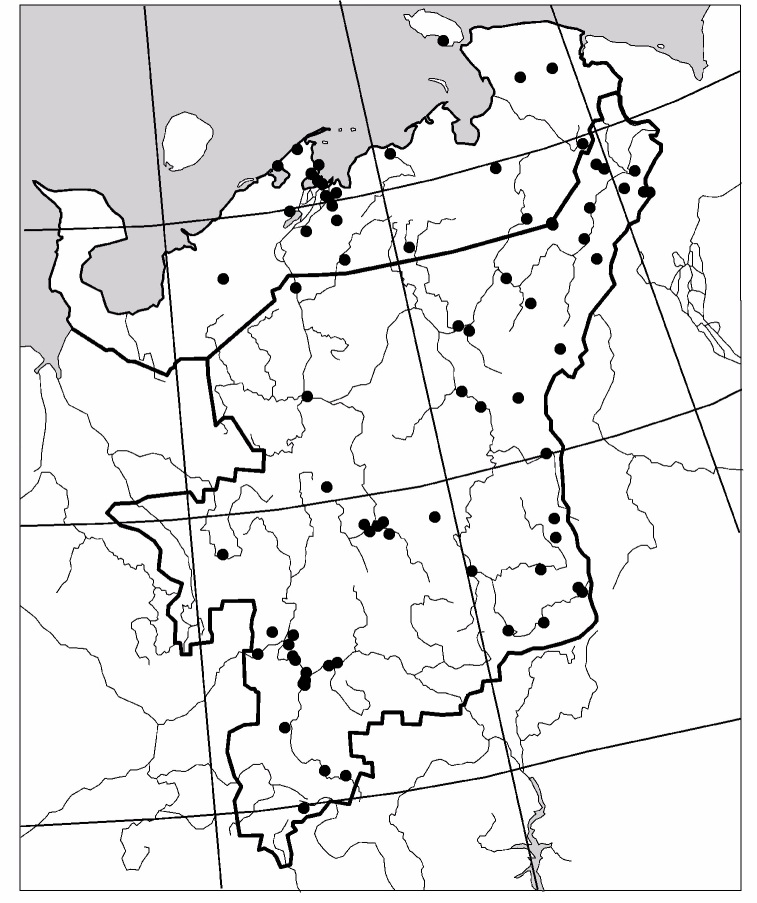
Sampling sites of Myriapoda in North-East Europe are included in the dataset.

**Figure 5.**
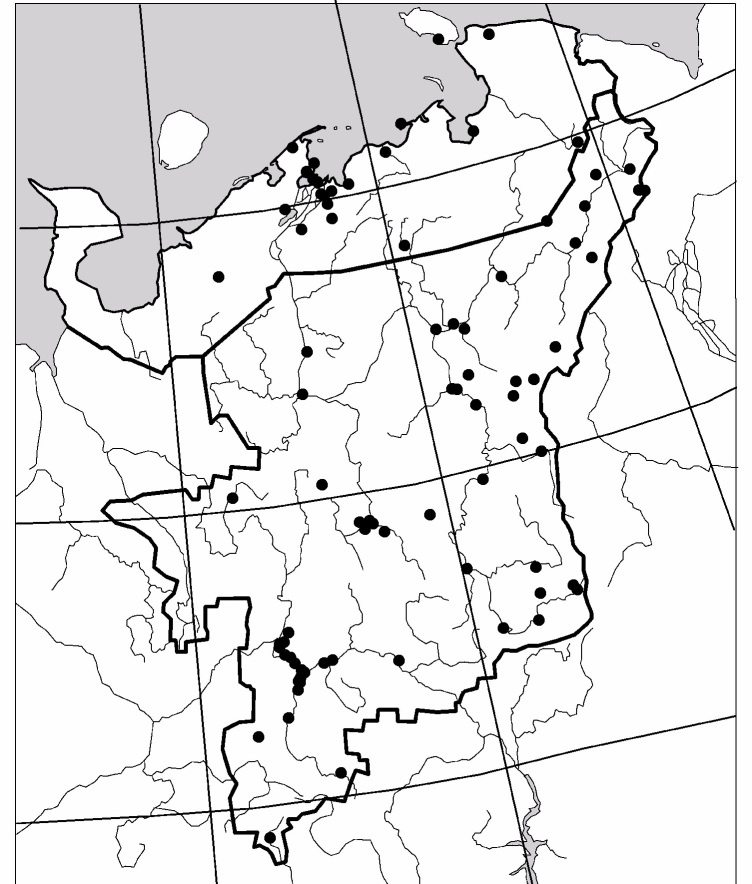
Sampling sites of Staphylinidae in North-East Europe are included in the dataset.

**Figure 6.**
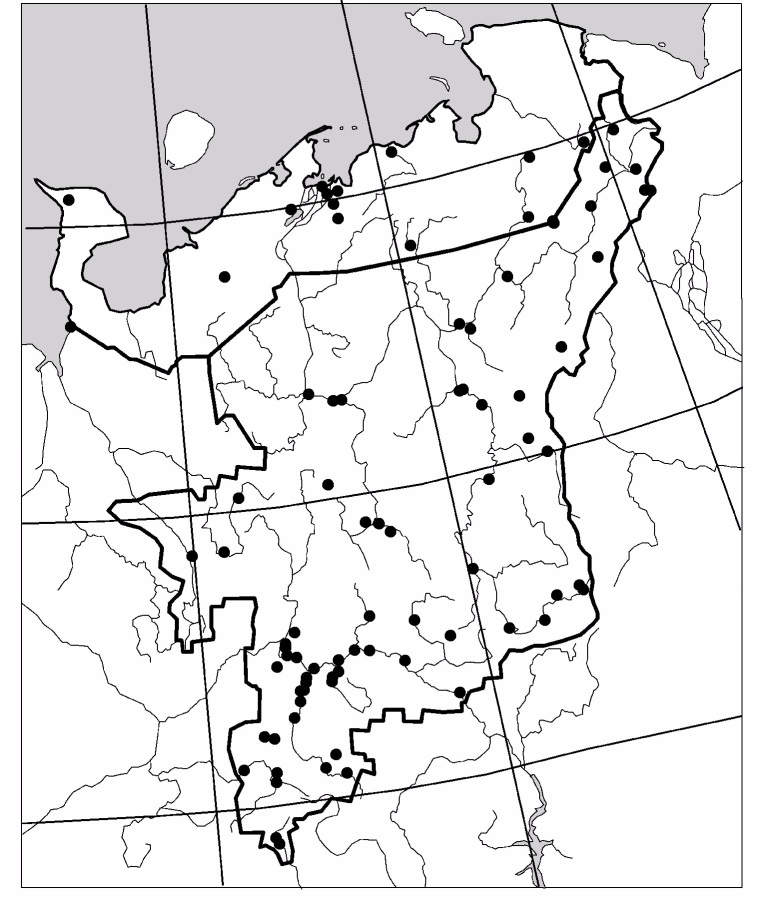
Sampling sites of Elateridae in North-East Europe are included in the dataset.

**Figure 7.**
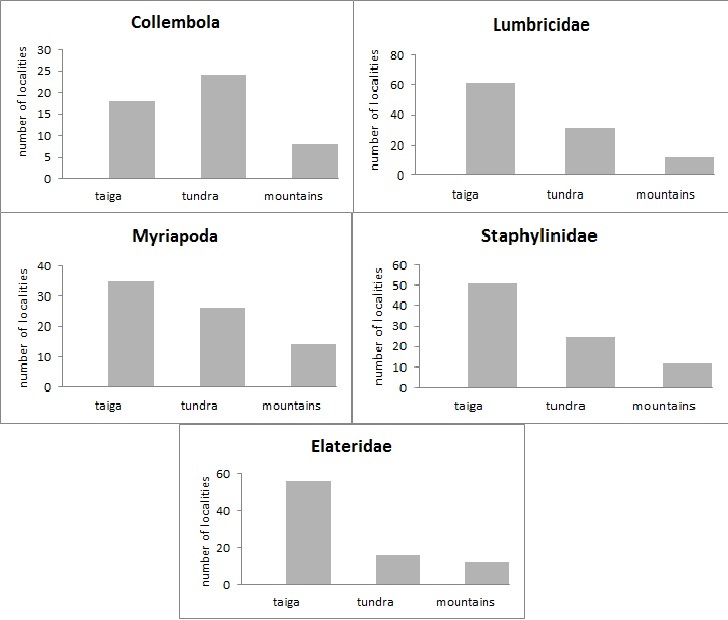
Number of records included in the datasets for each typology of habitat.

The study was carried out in the European part of Russia (Fig. 1). This dataset comprises data for 50 localities of Collembola, 104 – of Lumbricidae, 79 –Myriapoda, 88 – Staphylinidae and 84 – Elateridae (Figs 2, 3, 4, 5, 6). Amongst the reviewed groups, earthworms, millipedes, rove beetles and click beetles are better studied in the taiga zone. However, it is worth noting that the majority of springtail records are from tundra (Fig. 7).

### Coordinates

56.4 and 70.37 Latitude; 44.06 and 65.29 Longitude.

## Taxonomic coverage

### Description

All invertebrates were identified to species.

**Figure 8.**
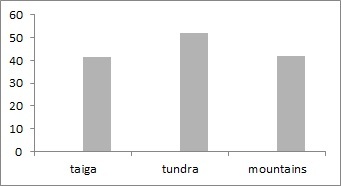
Springtail species richness in localities of the European North-East of Russia dataset.

European North-East of Russia localities, included in the dataset, account for over 246 springtail species. In the world, there are more than 8.6 thousand species of springtails, but due to the description of new taxa, this number is constantly increasing (Zhang et al. 2018). The number of Collembola species in tundra localities ranges from 10 to 105 (mean = 52.2, s.d. = 28.3), in taiga - from 9 to 80 (mean = 41.5, s.d. = 20.8) and in mountains ecosystems – from 12 to 62 (mean = 42.1, s.d. = 19.5, Fig. 8). Most of the species in the dataset are recorded from tundra as well as the majority of localities studied here. Altogether, these species account for 2,326 unique geo-referenced occurrence records.

**Figure 9.**
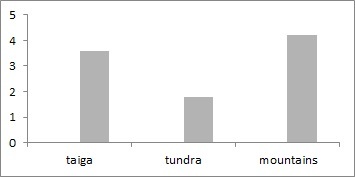
Earthworms species richness in localities of the European North-East of Russia dataset

European North-East of Russia localities included in the dataset account for over 13 species of earthworms. The world earthworm fauna contains 3.7 thousand species (Hendrix et al. 2008), while only 50 species are known for Russia (Vsevolodova-Perel 1997). The lowest values of species richness occurred across the boreal/subarctic regions, which was expected, based on aboveground biodiversity patterns (Phillips et al. 2019). This low earthworm diversity could be due to these regions typically being outside of the optimal temperature range (12-20˚C) for earthworms (Reynolds 1994). The number of earthworms species in tundra localities varies frrom 1 to 5 (mean = 1.8, s.d. = 0.2), in taiga – 1-10 (mean = 3.6, s.d. = 0.3) and the Urals – from 1 to 10 (mean = 4.2, s.d. = 0.6, Fig. 9). In East European tundra, *D. octaedra* and *E. nordenskioldi nordenskioldi* are well distributed and have a number of adaptations to their existence in harsh northern conditions (Makarova and Kolesnikova 2019). *L. rubellus* is met very seldom and two species are found only in anthropogenic soils (Kolesnikova et al. 2019). It is noticed that, in the Urals, the average number of species of worms is higher than in the taiga and tundra ecosystems (Fig. 4), since here, in addition to the typical inhabitants of taiga and tundra landscapes, there are endemics *Perelia diplotetratheca* and *Eisenia atlavinitea*. Altogether, these species account for 329 unique geo-referenced occurrence records.

**Figure 10.**
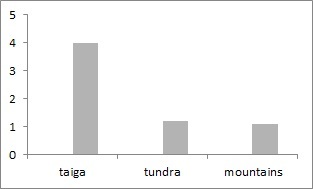
Millipedes species richness in localities of the European North-East of Russia dataset.

More than 3100 species of Chilopoda (Edgecombe et al. 2010) and more than 11000 species of Diplopoda (Enghoff et al. 2015) are known in the world fauna. We reveal 20 species of millipedes for European North-East of Russia.The largest number of species is noticed in the taiga zone, where its value ranges from 1 to 10 (mean = 4, s.d. = 0.5). In tundra, it varies from 1 to 4 (mean = 1.2, s.d. = 0.1) and, in mountains, only 1-2 (mean = 1.1, s.d. = 0.1) species are encountered (Fig. 10). This trend is natural as the *Lithobius curtipes* is the dominant species. Altogether, these species account for 202 unique geo-referenced occurrence records.

**Figure 11.**
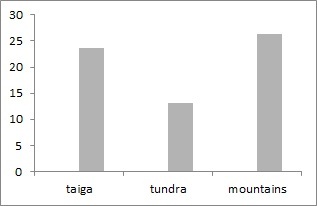
Rove beetles species richness in localities of the European North-East of Russia dataset.

Staphilinids are one of the most extensive and diverse families amongst beetles with about 1500 genera and more than 60000 species known in the world fauna and at least 4000 species in Russia (Herman 2001). European North-East of Russia localities, included in the dataset, account for over 446 species of rove beetles, that is only 11% of the Staphilinids diversity of Russia. The number of species per locality ranges from 1 to 76 (mean = 13.1, s.d. = 3) in tundra, from 1-140 (mean = 23.6, s.d. = 4.3) in taiga and from 2 to 58 (mean = 26.4, s.d. = 5.3) in mountains (Fig. 11). In tundra landscapes, the cold-resistant Omaliinae and Aleocharinae prevail. The slight excess of the average species richness in the Urals mountains compared to the taiga zone is explained by the high-altitude zone of the mountains, where species of boreal and arctic fauna inhabiting the mountain-forest and mountain-tundra belts, as well as the Ural-Siberian species, such as *Tachinus bicuspidatus*, are registered. Altogether, these species account for 1829 unique geo-referenced occurrence records.

**Figure 12.**
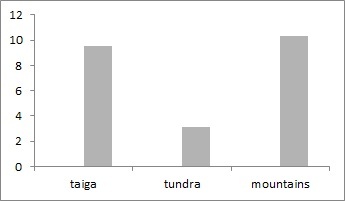
Click beetles species richness in localities of the European North-East of Russia dataset.

The world fauna of click beetles includes more than 10000 species (Costa et al. 2010). Amongst them, only 350 species are known for Russia (Prosvirov 2013). Our data include 60 species, i.e. more than 17% of their diversity of Russia. Elateridae are also better studied in the taiga zone, where the species richness ranges from 1 to 39 species (mean = 9.5, s.d. = 1.6). In the tundra ecosystems, the number of species is minimal and varies from 1 to 14 species (mean = 3.1, s.d. = 0.9). The fauna is presented by small representatives of the genera Hypnoidus and Oedostethus. Actually, only one species *Hypnoidus rivularis* is constantly found. It is numerous and inhabits a wide variety of types of tundra: shrubs, grains, moss-lichen and willow (Medvedev 2005). The average number of species in the Urals is slightly higher than in the taiga, due to inhabitants of mountain ecosystems, such as *Selatosomus gloriosus* (Fig. 12). Altogether, these species account for 727 unique geo-referenced occurrence records.

### Taxa included

**Table:** 

Rank	Scientific Name	
phylum	Annelida	
class	Clitellata	
order	Crassiclitellata	
family	Lumbricidae	
phylum	Arthropoda	
class	Chilopoda	
class	Diplopoda	
order	Collembola	
order	Coleoptera	
family	Staphylinidae	
family	Elateridae	

## Traits coverage

### Data coverage of traits

PLEASE FILL IN TRAIT INFORMATION HERE

## Temporal coverage

### Notes

Data sources provided the dates when the species was detected for the first time in a given region of the 5412 records included in the dataset. The earliest first record dates back to 1905 and the most recent event occurred in 2019.

## Collection data

### Collection name

Soil invertebrates occurrences in European North-East of Russia

### Specimen preservation method

alcohol, formalin, dried

## Usage licence

### Usage licence

Other

### IP rights notes

This work is licensed under a Creative Commons Attribution Non Commercial (CC-BY-NC) 4.0 Licence.

## Data resources

### Data package title

Soil invertebrates occurrences in European North-East of Russia

### Resource link

http://ib.komisc.ru:8088/ipt/resource?r=inv

### Alternative identifiers

https://www.gbif.org/dataset/a3f7d0d8-d9e1-42ae-b1f7-d1fc445b931d; https://doi.org/10.15468/5a8ydf

### Number of data sets

1

### Data set 1.

#### Data set name

Soil invertebrates occurrences in European North-East of Russia

#### Data format

Darwin Core

#### Number of columns

20

#### Download URL

http://ib.komisc.ru:8088/ipt/resource?r=inv

#### Description

The current project surveys 13 species of earthworms, 20 species of millipedes, 246 species of springtails, 446 species of rove beetles and 60 species of click beetles. A total of 5412 occurrences are included in the resource (Konakova et al. 2020). The earliest first record dates back to 1905 and the most recent event occurred in 2019.

**Data set 1.** 

Column label	Column description
occurrenceID	An identifier for the Occurrence (as opposed to a particular digital record of theoccurrence).
recordedBy	A person, group or organisation responsible for recording the original Occurrence.
verbatimLocality	Original description of the place of the find.
year	The four-digit year in which the Event occurred, according to the Common Era Calendar.
decimalLongitude	The geographic longitude (in decimal degrees, using the spatial reference system given in geodeticDatum) of the geographic centre of a Location.
decimalLatitude	The geographic latitude (in decimal degrees, using the spatial reference system given in geodeticDatum) of the geographic centre of a Location.
stateProvince	Administrative-territorial unit of the country (oblast, okrug or republic)
county	The name of the country or major administrative unit in which the Location occurs.
associatedReferences	A list (concatenated and separated) of identifiers (publication, bibliographic reference, global unique identifier, URI) of literature associated with the Occurrence.
basisOfRecord	Recommended best practice is to use the standard label of one of the Darwin Core classes.
georeferencedBy	A list (concatenated and separated) of names of people, groups or organisations who determined the georeference (spatial representation) for the Location.
geodeticDatum	The ellipsoid, geodetic datum or spatial reference system (SRS) upon which the geographic coordinates given in decimalLatitude and decimalLongitude are based.
kingdom	The full scientific name of the kingdom in which the taxon is classified.
phylum	The full scientific name of the phylum in which the taxon is classified.
class	The full scientific name of the class in which the taxon is classified.
order	The full scientific name of the oder in which the taxon is classified.
family	The full scientific name of the family in which the taxon is classified.
genus	The full scientific name of the genus in which the taxon is classified.
scientificNameAuthorship	The authorship information for the scientificName formatted according to the conventions of the applicable nomenclaturalCode.
scientificName	The full scientific name, with authorship and date information, if known. When forming part of an Identification, this should be the name in the lowest level taxonomic rank that can be determined. This term should not contain identification qualifications, which should instead be supplied in the IdentificationQualifier term.
